# Development and psychometric properties of a belief-based Physical Activity Questionnaire for Diabetic Patients (PAQ-DP)

**DOI:** 10.1186/1471-2288-10-104

**Published:** 2010-11-09

**Authors:** Zeinab Ghazanfari, Shamsaddin Niknami, Fazlollah Ghofranipour, Ebrahim Hajizadeh, Ali Montazeri

**Affiliations:** 1 Department of Health Education, Tarbiat Modares University, Tehran, Iran; 2 Department of Public Health, Ilam University of Medical Sciences, Ilam, Iran; 3 Department of Biostatistics, Tarbiat Modares University, Tehran, Iran; 4 Department of Mental Health, Institute for Health Sciences Research, ACECR, Tehran, Iran

## Abstract

**Background:**

This study carried out to develop a scale for assessing diabetic patients' perceptions about physical activity and to test its psychometric properties (The Physical Activity Questionnaire for Diabetic Patients-PAQ-DP).

**Methods:**

An item pool extracted from the Theory of Planned Behavior literature was generated. Then an expert panel evaluated the items by assessing content validity index and content validity ratio. Consequently exploratory factor analysis (EFA) was performed to indicate the scale constructs. In addition reliability analyses including internal consistency and test-retest analysis were carried out.

**Results:**

In all a sample of 127 women with diabetes participated in the study. Twenty-two items were initially extracted from the literature. A six-factor solution (containing 19 items) emerged as a result of an exploratory factor analysis namely: instrumental attitude, subjective norm, perceived behavioral control, affective attitude, self-identity, and intention explaining 60.30% of the variance observed. Additional analyses indicated satisfactory results for internal consistency (Cronbach's alpha ranging from 0.54 to 0.8) and intraclass correlation coefficients (ranging from 0.40 to 0.92).

**Conclusions:**

The Physical Activity Questionnaire for Diabetic Patients (PAQ-DP) is the first instrument that applies the Theory of Planned Behavior in its constructs. The findings indicated that the PAQ-DP is a reliable and valid measure for assessing physical activity perceptions and now is available and can be used in future studies.

## Background

Diabetes mellitus (DM) is an increasingly global public health problem. The number of people (aged 20-79 years) affected by diabetes in world will increase from 6.4% (285 million adults) in 2010 to 7.7%, (439 million adults) by 2030. This increase will be 69% in developing countries while developed countries will experience a 20% increase during the same period [1].

Physical activity reduces the risk of over 25 chronic conditions, in particular, coronary heart disease, stroke, hypertension, breast cancer, colon cancer, osteoporosis, and type 2 diabetes [2]. Regular physical activity is recommended for type 2 diabetic patients, as it is commonly known to correct metabolic disorders and prevent complications such as cardiovascular diseases [3]. The Canadian Diabetes Association's (CDA) Clinical Practice Guidelines for the prevention and management of diabetes recommended that type 2 diabetic patients should participate in moderate-intensity physical activity, such as brisk walking and biking, for at least 150 minutes/week, over at least 3 nonconsecutive days [4]. However recent studies have shown that diabetic patients may undertake less physical activity than non-diabetic people. Up to one third of diabetic patients are completely sedentary, and only a third exercise regularly [5]. To change this to a more healthy behavior the need for theory-driven interventions are recommended [6,7]. Theory provides a road map for studying problems, developing appropriate interventions, and evaluating their successes. Theory can also help planners identify the most suitable target audiences, methods for fostering change, and outcomes for evaluation [6]. The Theory of Planned Behavior (TPB) [8] is a well-established theory that has been used to further understand predictive factors of health behaviors in general [9,10] and physical activity in particular [11-14]. Numerous studies have provided support for the TPB to explain why some people practice physical activity and some do not [15-19].

This theory suggests that people's intention to perform a specific behavior is predicted or influenced by three determinants: attitudes, subjective norms and perceived behavioral control. Attitude refers to a personal factor of like or dislike, subjective norms refers to an individual's perception of social pressure, and perceived behavioral control refers to a person's perceived confidence in the ability to perform a behavior [20]. Hagger et al. reviewed 79 studies in a meta-analysis and reported that the TPB explained 44.5% of the variance in physical activity intentions and 27.4% of the variance in physical activity behavior [14].

The application of TPB in studies of physical activity in diabetic patients are well documented. For instance Plotnicoff et al. in a study on diabetic patients found the TPB constructs explained almost 40% of the variance in intentions for type 1 and type 2 diabetes. In cross-sectional studies, the TPB accounted for 23 and 19% of the variance in PA for type 1 and type 2 diabetes, respectively. In prospective studies, the TPB explained 13 and 8% of the variance in PA for type 1 and type 2 diabetes, respectively. The findings provide evidence for the utility of the TPB for the design of PA promotion interventions for adults with either type 1 or type 2 diabetes [21]. Also Davies et al. found that intention explained 28% of the variance in physical activity behavior. Attitude, subjective norm and perceived behavioral control (PBC) explained 73% of variance in physical activity intention. Attitude and PBC mediated the relationship between conscientiousness and physical activity intention. These results showed that targeting constructs proximal to the behavior (attitudes and PBC) may be effective in overcoming inherent qualities such as personality in order to produce physical activity behavior change within this sample population [22]. Data show links between sedentary behavior and all-cause mortality, cardiovascular disease, obesity, and adverse metabolic profiles [23]. Preliminary findings from STIL Project suggest that inactivity is more complex than we sometimes think [24]. Thus, in order to develop effective interventions to promote habitual physical activity, the predictive factors of this behavior is needed to be identified [25].

At present there are no comprehensive validated scales for measuring diabetic patients' beliefs regarding physical activity. Such instruments could help to understand patients' perspectives and in turn design comprehensive interventional programs for diabetic patients. Available instruments review particular aspects of the beliefs such as perceived benefits and barriers [26-31], outcome expectancy [29-32], self-efficacy [30,33], social support [29,30], enjoyment of physical activity [29,30], social influences [33], that were developed in the framework of other health belief models. There was one more questionnaire, developed by Blue et al., assesses the indirect measures of the theory of planned behavior including behavioral beliefs, normative beliefs and control beliefs to predict physical activity intentions of persons at risk for diabetes [34]. No questionnaire was found in the context of direct measures of the Theory of Planned Behavior. The main objective of this study was to design and develop an instrument in such a framework. However, this study included an additional construct (self-identity) to the original theory. As Ajzen suggests the Theory of Planned Behavior is open for further extension with additional constructs [35]. Studies have shown that individuals who identify themselves as exercisers have more favorable intentions and engage significantly more in exercise than those who do not [36-38]. It is argued that self-identity may play an important role in predicting physical activity [36-38]. Furthermore, this study was limited to a sample of female diabetic patients. It is estimated that more than 1.5 million people with diabetes live in Iran. The preliminary results derived from a national study indicated that the prevalence of type 2 diabetes was 3.6% among adults aged over 30 (4.3% of women and 2.6% of men) [39]. The figures clearly indicate that, as many other countries, women in Iran are suffering more from diabetes. In addition, the existing data from Iran indicate that prevalence of sedentary lifestyle in females is higher than males [40-42]. Sarrafzadegan et al. found that abdominal obesity was nearly six times as prevalent in women as in men (71.7% vs. 12%, P < 0.05) [43].

## Methods

### Definition and measure

Physical activity was defined as activities with moderate intensity, at least 3 or more times per week, accumulating at least 150 minutes per week derived from Health Canada's position stand for recommended weekly exercise among diabetic adults [4]. Physical activity was assessed by the Seven Day Physical Activity Recall Questionnaire [44,45].

### Scale development

First, we generated an item pool extracted from the literature [25,46-68] relating to the Theory of Planned Behavior (Table 1). The initial scale consisted of 22 items. Each item was rated on a 5-point Likert scale anchored at 1 to 5 (strongly agree to strongly disagree, very likely to very unlikely, very beneficial to very harmful, very worthwhile to very worthless, very good to very bad, very enjoyable to very boring, very relaxing to very stressful, strongly satisfied to strongly unsatisfied) Table 2.

**Table 1:** The list of papers for generating item pool (derived from Theory of Planned Behavior (TPB) literature)

Author(s)	Year	No. of items	Constructs
Courneya, et al. [46]	1998	16	Attitude, subjective norm, perceived behavioral control, intention

Courneya et al. [47]	1999	15	Attitude, subjective norm, perceived behavioral control, intention

Courneya, & Bobick [48]	2000	17	Attitude, subjective norm, perceived behavioral control, intention

Blue, et al [49]	2001	12	Attitude, subjective norm, perceived behavioral control, behavioral, normative and control beliefs, intention

Jackson, et al. [25]	2003	27	Attitude, subjective norm, perceived behavioral control, intention, descriptive norm, personal norm (moral norm and anticipated affective reaction), self-identity, past behavior

Rhodes, & Courneya [50]	2003	13	Attitude, subjective norm, perceived behavioral control, intention

Francis, et al [51]	2004	26	Attitude (instrumental, affective), subjective norm (injunctive, descriptive), perceived behavioral control (self-efficacy, controllability), behavioral, normative and control beliefs, intention (generalized, statement)

Hausenblas, & Downs [52]	2004	13	Attitude, subjective norm, perceived behavioral control, intention

Rhodes, & Courneya [53]	2004	21	Attitude, subjective norm, perceived behavioral control, intention

Prapavessis, et al. [54]	2005	19	Attitude, subjective norm, perceived behavioral control, intention

Rhodes, & Plotnikoff [55]	2005	9	Attitude, subjective norm, perceived behavioral control, intention

Walsh, et al. [56]	2005	11	Attitude, subjective norm, perceived behavioral control, intention

Ajzen I [57]	2006	26	Attitude (instrumental, affective), subjective norm (injunctive, descriptive), perceived behavioral control (capability, controllability), behavioral, normative and control beliefs, intention

Courneya, et al. [58]	2006	21	Instrumental and affective attitudes, injunctive and descriptive norms, perceived control, intention

Kaiser [59]	2006	12	Attitude, subjective norm, perceived behavioral control, intention

White, et al. [60]	2007	14	Behavioral, normative and control beliefs

Hill, et al. [61]	2007	9	Attitude, normative beliefs, perceived behavioral control, intention

Blue [62]	2007	45	Attitude, subjective norm, perceived behavioral control, intention, behavioral, normative and control beliefs

Jones, et al. [63]	2007	14	Attitude, subjective norm, perceived behavioral control, intention

Plotnikoff, et al. [64]	2008	14	Attitude, subjective norm, perceived behavioral control, intention

Parrott, et al. [65]	2008	12	Instrumental and affective attitudes, subjective norm, perceived behavioral control, intention

Chatzisarantis, et al. [66]	2008	14	Attitudes, subjective norms, perceived behavioral control, intention

Boudreau, & Godin [67]	2009	45	Attitude, subjective norm, perceived behavioural control, intention, behavioural beliefs, normative beliefs, control beliefs, anticipated regret, moral norm, descriptive norm

**Table 2:** The PAQ-DP items extracted from the literature in the context of Theory of Planned Behavior

	Item Pools	Response categories
1	Doing 30 minutes of moderate physical activity at least 5 days a week would be .....	Very beneficial - beneficial - neither beneficial nor harmful - harmful - very harmful

2	Doing 30 minutes of moderate physical activity at least 5 days a week would be ....	Very worthwhile - worthwhile - neither worthwhile nor worthless - worthless - very worthless

3	Doing 30 minutes of moderate physical activity at least 5 days a week would be .....	Very good - good - neither good nor bad - bad - very bad

4	Doing 30 minutes of moderate physical activity at least 5 days a week would be .....	Very enjoyable- enjoyable -neither enjoyable nor boring - boring -very boring

5	Doing 30 minutes of moderate physical activity at least 5 days a week would be .....	Very relaxing - relaxing - neither relaxing nor stressful - stressful - very stressful

6	People who are important to me think I should do 30 minutes of moderate physical activity at least 5 days a week.	Strongly agree - agree - neither agree nor disagree - disagree -strongly disagree

7	People who are important to me want me to do 30 minutes of moderate physical activity at least 5 days a week.	Strongly agree - agree - neither agree nor disagree - disagree - strongly disagree

8	People who are important to me would expect me to do 30 minutes of moderate physical activity at least 5 days a week.	Strongly agree - agree - neither agree nor disagree - disagree - strongly disagree

9	For me to do 30 minutes of moderate physical activity at least 5 days a week is difficult	Strongly agree - agree - neither agree nor disagree - disagree - strongly disagree

10	Doing 30 minutes of moderate physical activity at least 5 days a week is up to me	Strongly agree - agree - neither agree nor disagree - disagree - strongly disagree

11*	If I want, I can to do 30 minutes of moderate physical activity at least 5 days a week	Strongly agree - agree - neither agree nor disagree - disagree - strongly disagree

12*	I am going to do 30 minutes of moderate physical activity on .... day (days)	0, 1-2, 3, 4, 5-7

13	How likely is it possible that you would make a decision to do 30 minutes moderate physical activity at least 5 days a week in the next month?	Very likely - likely - neither likely nor unlikely - unlikely - very unlikely

14	I expect to do 30 minutes of moderate physical activity at least 5 days a week	Very likely - likely - neither likely nor unlikely - unlikely - very unlikely

15*	The most of important others for me do 30 minutes of moderate physical activity at least 5 days a week	Strongly agree - agree - neither agree nor disagree - disagree - strongly disagree

16	I would feel sick about not doing 30 minutes of moderate physical activity at least 5 days a week	Strongly agree - agree - neither agree nor disagree - disagree - strongly disagree

17	I am under pressure from my family or friends to do 30 minutes of moderate physical activity at least 5 days a week	Strongly agree - agree - neither agree nor disagree - disagree - strongly disagree

18	Doing 30 minutes of moderate physical activity at least 5 days a week would make me ....	Strongly satisfied - satisfied - neither satisfied nor unsatisfied - unsatisfied - strongly unsatisfied

19	I see myself as sporty	Strongly agree - agree - neither agree nor disagree - disagree - strongly disagree

20	I see myself as fit and healthy	Strongly agree - agree - neither agree nor disagree - disagree - strongly disagree

21	I see myself as a physically active person	Strongly agree - agree - neither agree nor disagree - disagree - strongly disagree

22	Others might see me as a couch potato	Strongly agree - agree - neither agree nor disagree - disagree - strongly disagree

* Deleted items in the final version.

Subsequently an expert panel of 10 specialists in health education, diabetes, and physical education examined the initial questionnaire. The panel was asked to comment on individual items in relation to the accuracy, clarity, and style. Items were slightly modified based on expert reviews. Then, a different panel of 11 experts on health education was asked to comment independently on *necessity *and *relevance *of the items in order to calculate Content Validity Ratio (CVR) and Content Validity Index (CVI), respectively. The necessity of the items was assessed using a three-point rating scale: (i) not necessary, (ii) useful, but not essential, (iii) essential. Following the experts' assessments, a CVR for total scale was computed. According to Lawshe, if more than half of the panelists indicate that an item is essential, that item has at least content validity [69]. The CVR in this study for total scale was .61 indicating a satisfactory result. The relevance of the items was also assessed using a four-point rating scale: (i) not relevant, (ii) slightly relevant, (iii) relevant, and (iv) very relevant. The CVI of each question is the proportion of experts who rate it as 3 or 4 [70]. Polite and Beck recommended 0.80 for the acceptable lower limit for CVI value [71]. A satisfactory level of agreement was found (CVI = 0.91) among panelists suggesting that the scale had a good content validity.

### Face validity

The provisional scale was then administered to a sample of 5 diabetic women with different socio-demographic characteristics in order to assess clarity and readability of the items. In general there were no major problems in reading and understanding the items by women. However, a few words were changed to meet women's considerations.

### The study and participants

In order to test the scale in a wider context, a cross-sectional study carried out in the Charity Foundation for Special Diseases (CFFSD), in Tehran, Iran between November 2008 and July 2009. The inclusion criteria were: aged between 15 to 70 years, being literate, no history of diabetes complications, and mental and disabling disorders. To collect data, trained interviewers carried out face-to-face interviews.

### Statistical analysis

#### Validity

*1. Construct validity: *The dimensionality of the scale was determined by performing exploratory factor analysis (EFA) using the principal axis factoring and oblique rotation. Since correlation between factors was less than 0.3, varimax rotation with Kaiser Normalization was selected [72]. In order to evaluate sampling adequacy to perform a satisfactory factor analysis, Kaiser-Meyer-Olkin Measure of Sampling Adequacy (KMO) and Bartlett test was calculated. To determine the best structure, the eigenvalue greater than one and factor loading equal to or greater than 0.4 were applied [73].

*2. Convergent validity: *convergent validity was assessed performing item-scale correlations corrected for overlaps. Correlations were calculated using Pearson' correlation coefficient. It was expected that item scores would correlate higher with own hypothesized scale than other scales. Correlation values of 0.40 or above were considered satisfactory (r ≥ 0.81-1 as excellent, 0.61-0.80 very good, 0.41-0.60 good, 0.21-0.40 fair, and 00-0.20 poor) [74].

#### Reliability

*1. Internal consistency: *The internal consistency of PAQ-DP was estimated by computing Cronbachs' alpha coefficient. The alpha values of 0.70 or above were considered satisfactory [74].

*2. Test-retest: *The test-retest reliability of the scale was estimated by intraclass correlation coefficient (ICC). The scale was re-administered to 16 individuals 1 month after the first completion. The following category was selected to interpret the agreement levels: 00-0.2 as slight, 0.21-0.40 as fair, 0.41-0.60 as moderate, 0.61-0.80 as substantial, and 0.81-1 as almost perfect [75].

#### Scoring

The final version of the questionnaire is available [Additional file 1]. In addition details of scoring are provided. However, in summary each item is scored from 5 to 1 to provide row scores. Since three items on perceived difficulty, and capability of doing physical activity, and others' perceptions about one's mobility were negatively worded, scoring for these items (items 9, 10, and 22 in first version and 9, 10, and 19 in the final version) was reversed. A linear transformation was used to calculate scores ranging from 0 to 100; where higher scores indicate better perceptions about physical activity [Additional file 2].

#### Ethical considerations

The Ethics Committee of Tarbiat Modares University approved the study. All participants gave their permission by signing an informed consent form.

## Results

A total of 127 diabetic patients participated in the study. The mean age of participants was 46.40 (SD = ± 11.4) years. Most participants (86%) enjoyed secondary education and were married (81%). There were 109 patients with type 2 diabetes mellitus (86%) and the remaining 18 patients with had type 1 diabetes (14%). The results are shown in Table 3.

**Table 3:** Demographic and medical profile of the participants (n = 127)

		No. (%)
**Age**	Year, Mean (SD)	46.40 ± 11.40

**Education**		

	Primary	28 (22)

	Secondary	81 (64)

	Higher	18 (14)

**Marital status**		

	Single	12 (9.40)

	Married	103 (81)

	Divorced or Widowed	12 (9.40)

**Employment**		

	Housekeeper	101 (80.20)

	Employed	17 (13.50)

	Student	8 (6.30)

**Smoking status**		

	Yes	11 (8.70)

	No	116 (91.30)

**Diabetes type**		

	Type 1	18 (14.20)

	Type 2	109 (85.80)

**BMI**	Mean (SD)	27.80 ± 5

**Duration**	Year, Mean (SD)	8.50 ± 6

**Physical activity**	Min, Mean (SD)	38.08 (73.81)

Exploratory factor analysis (EFA) was used to determine the underlying factor structure of the set of items. The calculated KMO was 0.73 and the Bartlett's test of sphericity was significant (P < 0.0001). A six-factor solution with 19 items emerged based on eigenvalues higher than 1 and loading level of 0.4 or above. The six factors were named according to the underlying construct that related to the items: instrumental attitude, subjective norm, self-identity, perceived behavioral control, affective attitude, and intention. The six-factor solution explained 60.30% of the total variance of the hypothesized model. The detailed results are shown in Table 4.

**Table 4:** Results obtained from exploratory factor analysis

		F1	F2	F3	F4	F5	F6
1	Beneficial - harmful	**0.79**	0.08	-0.01	0.05	0.09	0.13

2	Worthwhile - worthless	**0.74**	0.09	0.18	-0.04	0.14	0.09

3	Good - bad	**0.84**	0.15	0.00	0.1	0.01	-0.03

4	Enjoyable - boring	0.45	0.08	0.15	0.20	-0.09	**0.48**

5	Relaxing - stressful	**0.53**	0.12	0.01	0.12	-0.01	0.47

6	Think	0.28	**0.68**	-0.07	0.02	0.33	-0.01

7	Want	0.21	**0.72**	0.05	-0.03	0.29	0.10

8	Expect	0.29	**0.63**	-0.01	-0.07	0.29	0.08

9	Capability of doing physical activity	0.06	0.02	0.09	**0.88**	0.08	0.00

10	Difficulty of doing physical activity	0.03	-0.03	0.17	**0.56**	0.15	0.14

11	If I want, I can	0.11	0.30	0.17	0.29	0.01	-0.04

12	I am going to do	0.05	-0.09	0.18	0.27	0.23	-0.01

13	How likely	0.00	0.12	0.10	0.16	**0.67**	0.10

14	I expect to do	0.13	0.17	0.12	0.12	**0.49**	0.18

15	Important others do	-0.06	0.18	0.04	0.00	-0.03	0.02

16	I feel sick	0.01	0.03	0.05	-0.02	0.08	**0.43**

17	I am under pressure	0.10	**0.57**	-0.13	0.05	-0.12	0.27

18	Satisfied - unsatisfied	0.14	0.23	0.05	0.06	**0.27**	**0.60**

19	Sporty	0.05	0.01	**0.68**	0.14	0.18	0.07

20	Healthy	-0.01	0.19	**0.65**	0.25	0.02	-0.03

21	Physically active person	0.05	0.06	**0.62**	0.03	0.10	0.01

22	Couch potato	0.05	-0.14	**0.48**	0.06	-0.03	0.13

	Eigenvalue	4.80	2.60	1.90	1.40	1.40	1.20

	Explained variance (%)	21.70	11.73	8.57	6.44	6.24	5.53

**F1**: Instrumental attitude, **F2**: Subjective norm, **F3**: Self-identity, **F4**: Perceived behavioral control, **F5**: Intention, **F6**: Affective attitude

Descriptive statistics including Cronbach's alpha, ICC, mean scores, and standard deviations for the PAQ-DP are presented in Table 5. The Cronbach's alpha for the subscales ranged from 0.54 to 0.82. The intraclass correlation coefficient for the PAQ-DP subscales was satisfactory (ICC ranged from 0.40 to 0.92). The correlation matrix is presented in Table 6. As expected the correlation between items belonging to any constructs of Theory of Planned Behavior was satisfactory.

**Table 5:** Descriptive statistics, Cronbach's alpha and intraclass correlation coefficient of the PAQ-DP subscales

	No of items	Mean (SD)*	Skewness	Cronbach' alpha	ICC**
Instrumental attitude	4	88.80 (11.80)	-1.70	0.82	0.79

Affective attitude	3	79.60 (13.70)	-0.80	0.54	0.56

Subjective norm	4	75.40 (16.50)	-0.70	0.76	0.92

Perceived behavioral control	2	58.10 (25.70)	-0.40	0.71	0.78

Self-identity	4	49.50 (20.20)	0.00	0.71	0.73

Intention	2	72 (16.50)	-0.40	0.60	0.40

*Scores range from 0 to 100. Higher scores indicate better perceptions about physical activity.

**Intraclass Correlation Coefficient

**Table 6:** Item-scale correlations of the Theory of Planned Behavior items and constructs

	Factors					
Factors	**IA**	**AA**	**SN**	**PBC**	**SI**	**Int**

**Instrumental attitude**	1	0.42**	0.39**	0.13	0.13	0.20*

Beneficial - harmful	0.84**	0.31**	0.33**	0.11	0.08	0.17

Worthwhile - worthless	0.82**	0.30**	0.30**	0.06	0.20*	0.19*

Good - bad	0.86**	0.22**	0.33**	0.12	0.06	0.13

Relaxing - stressful	0.73**	0.52**	0.31**	0.15	0.07	0.16

**Affective attitude**	0.42**	1	0.33**	0.18*	0.19*	0.25**

Enjoyable - boring	0.53**	0.64**	0.25**	0.23*	0.20*	0.10

I feel sick	0.13	0.73**	0.11	0.04	0.08	0.07

Satisfied - unsatisfied	0.30**	0.79**	0.37**	0.14	0.14	0.38**

**Subjective norm**	0.39**	0.33**	1	0.04	0.01	0.33**

Think	0.36**	0.23*	0.78**	0.07	-0.01	0.32**

Want	0.32**	0.28**	0.83**	0.05	0.11	0.33**

Expect	0.37**	0.24**	0.79**	-0.01	0.05	0.35**

I am under pressure	0.22*	0.27**	0.75**	0.02	-0.08	0.11

**Perceived behavioral control**	0.13	0.18*	0.04	1	0.28**	0.26**

Difficulty of doing physical activity	0.13	0.13	0.03	0.88**	0.23*	0.23**

Capability of doing physical activity	0.11	0.18*	0.04	0.89**	0.27**	0.23**

**Self-identity**	0.13	0.19*	0.01	0.28**	1	0.23*

Sporty	0.13	0.19*	0.01	0.21*	0.76**	0.26**

Healthy	0.07	0.11	0.12	0.31**	0.74**	0.16

Physically active person	0.10	0.09	0.04	0.15	0.75**	0.20*

Couch potato	0.07	0.15	-0.13	0.16	0.67**	0.04

**Intention**	0.20*	0.25**	0.33**	0.26**	0.23*	1

How likely	0.11	0.18*	0.25**	0.24**	0.19*	0.85**

I expect to do	0.23**	0.24**	0.31**	0.21*	0.20*	0.83**

*p < .05, **p < .01

**IA**: Instrumental attitude, **AA**: affective attitude, **SN**: Subjective norm, **PBC**: Perceived behavioral control, **SI**: Self-identity, **Int**: Intention,

## Discussion

The purpose of this study was to develop a scale for assessing women's perceptions about physical activity and to test its psychometric properties. This scale was developed based on Theory of Planned Behavior framework. In Theory of Planned Behavior, each predictor may be measured directly e.g. by asking respondents about their overall attitude, or indirectly e.g. by asking respondents about specific behavioral beliefs and its outcome. Usually for developing a TPB-based questionnaire, it has been suggested that for direct measures one should use the same direct measures developed by Ajzen [57] and Francis [51]. For indirect (belief-based) measures it has been recommended to carry out an elicitation study to develop all predictor constructs in the TPB that are attitude; subjective norm; and perceived behavioral control. Direct and indirect approaches make different assumptions about the underlying cognitive structures and neither approach is perfect. When different methods are tapping the same construct, scores are expected to be positively correlated, so it is recommended that both be included in a TPB-based questionnaire [51]. Unfortunately, in this study, only direct measures of TPB were used.

As Francis et al. suggested, each construct should be measured using a minimum of three items. This will result in a minimum of 12 items for intention and direct measures of the predicting variables [51]. However, in the primarily draft of the questionnaire presented in this study, 22 items included. In fact according to Ajzen and Francis [51,57], attitude, subjective norm, and perceived behavioral control each contained two subscales (attitude: instrumental and affective; subjective norm: injunctive and descriptive; and perceived behavioral control: self-efficacy and controllability).

Exploratory factor analysis was led to remove three items from a total of original 22 items and the final 19-item scale categorized in six factors explaining 60.3% of the variance observed. EFA with varimax rotation indicated that six factors including instrumental attitude, subjective norm, perceived behavioral control, affective attitude, self-identity, and intention can be extracted. As expected attitude was extracted with two subscales (instrumental and affective) but subjective norm and perceived behavioral control did load as single factors and expected subscales were not emerged separately.

Overall the internal consistency of the PAQ-DP was acceptable, although Cronbach's alpha for some dimensions were lower than 0.70. However, there was no significant increase in the Cronbachs' alpha when any items were removed. This result may be due to the fact that fewer items were included in these dimensions. Also, small sample size, high homogeneity of the patients, and small variability of the scores might decreased alpha coefficients [71]. In addition, as Ajzen suggested, adapting items used in previous studies might produce measures with relatively low reliabilities and lead to an underestimate of the relations among the theory's constructs and of its predictive validity [57]. It seems that increasing the sample size, and increasing the number of items in some dimensions could further confirm the reliability of the scale. This result is very similar to those previously reported by Tan et al. [76] and Leung et al. [77].

There are some practical implications for developing a scale to measure diabetic patients' beliefs toward regular physical activity. Valid and reliable instruments are needed for designing and evaluating health programs based on behavior change theories of social and behavioral sciences [78]. Interventions to promote regular physical activity can target the beliefs that guide behavior. In other words, identifying beliefs could lead to development of effective programs and perhaps help to achieve healthy lifestyle for target populations. Health care providers who work with diabetic patients can use the measures to identify beliefs that promote regular physical activity and in turn intervene to delay or prevent diabetes complications. The PAQ-DP also could be a useful instrument for evaluating intervention effects by comparing the scores before and after patients' receiving various interventions; and for explaining the correlations of beliefs of diabetic patients with their exercise behavior.

The findings from this study should be interpreted with caution. This study had several inherent limitations: only female engaged in the study, sample size was relatively small, the reliability coefficients of some factors were not satisfactory. Also, only direct measures were used in the study. In addition 6 factors and 19 items means that most factors might not be quite deep. The future studies using this questionnaire could help to overcome these problems.

## Conclusion

Overall, the study findings suggest that the PAQ-DP is a valid and reliable instrument for assessing beliefs of female diabetic patients regarding physical activity. The findings of the current study support the Ajzen's Theory of Planned Behavior. Further studies are recommended to confirm its application in clinical practice.

## Competing interests

The authors declare that they have no competing interests.

## Authors' contributions

ZGh was the main investigator, collected the data, performed the statistical analysis, and drafted the manuscript. SHN supervised the research and contributed to all aspects of the study. FGH was advisor of the study and contributed to study design. EH helped in statistical analysis. AM helped as a consultant in study design, questionnaire, and revised the final article. All authors read and approved the final manuscript.

## Pre-publication history

The pre-publication history for this paper can be accessed here:

http://www.biomedcentral.com/1471-2288/10/104/prepub

## Supplementary Material

Additional file 1 **Physical Activity Questionnaire for Diabetic Patients (PAQ-DP)**. This is the final version of the PAQ-DP that was developed by this study. The questionnaire could be used by other investigators providing that they cite this paper. Click here for file

Additional file 2 **Scoring Instruction for the PAQ-DP**. This is an instruction for scoring the PAQ-DP, Click here for file
