# Supplementary material for: Development and psychometric properties of a belief-based Physical Activity Questionnaire for Diabetic Patients (PAQ-DP)
Source: BMC Med Res Methodol. 2010 Nov 9;10:104. doi: 10.1186/1471-2288-10-104 (PMC2998522; doi:10.1186/1471-2288-10-104)
Supplement: Additional file 1 — Physical Activity Questionnaire for Diabetic Patients (PAQ-DP). This is the final version of the PAQ-DP that was developed by this study. The questionnaire could be used by other investigators providing that they cite this paper. [file 1471-2288-10-104-S1.DOC]

**Physical Activity Questionnaire for Diabetic Patients (PAQ-DP)**

ID: ………………

Date: …………….

**Instruction**

This questionnaire deals with perceptions that you have about being physically active. For each question, circle the answer that best describes your beliefs or feelings. Please answer all questions.

*Please note that in all instances moderate physical activity such as walking, swimming, biking, means those activities that increases your heartbeat a little and causes sweating.*

| 1. Doing 30 minutes of moderate physical activity at least 5 days a week would be ….. | |
| --- | --- |
| a) Very beneficial |  |
| b) Beneficial |  |
| c) Neither beneficial nor harmful |  |
| d) Harmful |  |
| e) Very harmful |  |
| 2. Doing 30 minutes of moderate physical activity at least 5 days a week would be …. | |
| a) Very worthwhile |  |
| b) Worthwhile |  |
| c) Neither worthwhile nor worthless |  |
| d) Worthless |  |
| e) Very worthless |  |
| 3. Doing 30 minutes of moderate physical activity at least 5 days a week would be …. | |
| a) Very good |  |
| b) Good |  |
| c) Neither good nor bad |  |
| d) Bad |  |
| e) Very bad |  |
| 4. Doing 30 minutes of moderate physical activity at least 5 days a week would be ….. | |
| a) Very relaxing |  |
| b) Relaxing |  |
| c) Neither relaxing nor stressful |  |
| d) Stressful |  |
| e) Very stressful |  |
| 5. People who are important to me think I should do 30 minutes of moderate physical activity at least 5 days a week. | |
| a) Strongly agree |  |
| b) Agree |  |
| c) Neither agree nor disagree |  |
| d) Disagree |  |
| e) Strongly disagree |  |
| 6. People who are important to me want me to do 30 minutes of moderate physical activity at least 5 days a week. | |
| a) Strongly agree |  |
| b) Agree |  |
| c) Neither agree nor disagree |  |
| d) Disagree |  |
| e) Strongly disagree |  |
| 7. People who are important to me would expect me to do 30 minutes of moderate physical activity at least 5 days a week | |
| a) Strongly agree |  |
| b) Agree |  |
| c) Neither agree nor disagree |  |
| d) Disagree |  |
| e) Strongly disagree |  |
| 8. I am under pressure from my family or friends to do 30 minutes of moderate physical activity at least 5 days a week. | |
| a) Strongly agree |  |
| b) Agree |  |
| c) Neither agree nor disagree |  |
| d) Disagree |  |
| e) Strongly disagree |  |
| 9. For me to do 30 minutes of moderate physical activity at least 5 days a week is difficult. | |
| a) Strongly agree |  |
| b) Agree |  |
| c) Neither agree nor disagree |  |
| d) Disagree |  |
| e) Strongly disagree |  |
| 10. Doing 30 minutes of moderate physical activity at least 5 days a week is up to me. | |
| a) Strongly agree |  |
| b) Agree |  |
| c) Neither agree nor disagree |  |
| d) Disagree |  |
| e) Strongly disagree |  |
| 11. How likely is it possible that you would make a decision to do 30 minutes moderate physical activity at least 5 days a week in the next month? (The next month could be changed to period you wish to ask from respondents in your study) | |
| a) Very likely |  |
| b) Likely |  |
| c) Neither likely nor unlikely |  |
| d) Unlikely |  |
| e) Very unlikely |  |
| 12. I expect to do 30 minutes of moderate physical activity at least 5 days a week. | |
| a) Very likely |  |
| b) Likely |  |
| c) Neither likely nor unlikely |  |
| d) Unlikely |  |
| e) Very unlikely |  |
| 13. Doing 30 minutes of moderate physical activity at least 5 days a week would be ….. | |
| a) Very enjoyable |  |
| b) Enjoyable |  |
| c) Neither enjoyable nor boring |  |
| d) Boring |  |
| e) Very boring |  |
| 14. I would feel sick about not doing 30 minutes of moderate physical activity at least 5 days a week. | |
| a) Strongly agree |  |
| b) Agree |  |
| c) Neither agree nor disagree |  |
| d) Disagree |  |
| e) Strongly disagree |  |
| 15. Doing 30 minutes of moderate physical activity at least 5 days a week would make me …. | |
| a) Strongly satisfied |  |
| b) Satisfied |  |
| c) Neither satisfied nor unsatisfied |  |
| d) Unsatisfied |  |
| e) Strongly unsatisfied |  |
| 16. I see myself as sporty. | |
| a) Strongly agree |  |
| b) Agree |  |
| c) Neither agree nor disagree |  |
| d) Disagree |  |
| e) Strongly disagree |  |
| 17. I see myself as fit and healthy. | |
| a) Strongly agree |  |
| b) Agree |  |
| c) Neither agree nor disagree |  |
| d) Disagree |  |
| e) Strongly disagree |  |
| 18. I see myself as a physically active person. | |
| a) Strongly agree |  |
| b) Agree |  |
| c) Neither agree nor disagree |  |
| d) Disagree |  |
| e) Strongly disagree |  |
| 19. Others might see me as a couch potato. | |
| a) Strongly agree |  |
| b) Agree |  |
| c) Neither agree nor disagree |  |
| d) Disagree |  |
| e) Strongly disagree |  |
| Would you like to add anything else? Please feel free and write below:  ……………………………………………………………………………………………………… | |
| **Thank you for completing the questionnaire.** | |

© Ghazanfari Z, et al., 2010
