# Supplementary material for: Development and psychometric properties of a belief-based Physical Activity Questionnaire for Diabetic Patients (PAQ-DP)
Source: BMC Med Res Methodol. 2010 Nov 9;10:104. doi: 10.1186/1471-2288-10-104 (PMC2998522; doi:10.1186/1471-2288-10-104)
Supplement: Additional file 2 — Scoring Instruction for the PAQ-DP. This is an instruction for scoring the PAQ-DP, [file 1471-2288-10-104-S2.DOC]

# Scoring Instruction for the PAQ-DP

The PAQ-DP includes six subscales namely: instrumental attitude (items 1-4), subjective norm (items 5-8), perceived behavioral control (items 9, 10), intention (items 11, 12), affective attitude (items 13-15), and self-identity (items 16-19).

To calculate the scores please fallow this instruction:

1. Score all items from 5 to 1, except for items 9, 10 and 19 where scoring should be reversed that is 1 to 5. These are row score for each item.

2. To calculate the row score for each subscale add item raw scores and then divide it to number of items in that subscale.

3. To transfer row scores to a score ranging from 0 to 100 then use the following formula to calculate the final score

The subscale score = ((subscale row score–1)/4) × 100

**Example 1:**

For example to calculate affective attitude (item 13, 14, and 15) for someone who scored item 1 as enjoyable, and item 2 as strongly agree and item 3 as unsatisfied, his/her score for each item would be 4, 5, and 2 respectively. The row score for this subscale then would be:

4+5+2/3: 3.33.

Then this should be linearly transformed:

((3.33-1)/4) × 100 = 58.25

**Example2:**

To calculate perceived behavioral control for someone who scored item 9 as agree and item 10 as disagree, his/her score for each item would be 2 and 4 respectively. The row score for this subscale then would be:

2+4/2: 3.0

Then this should be linearly transformed:

((3.0-1)/4) × 100 = 50
